# Whispers of Growth: The Impact of MicroRNA on the Proliferation Potential of Adipose‐Derived Stem Cells—A Review

**DOI:** 10.1155/bmri/4137354

**Published:** 2026-01-30

**Authors:** Maryam Bahrami, Hojjat Allah Abbaszadeh, Katayon Berjis, Ali Moradi

**Affiliations:** ^1^ Laser Applications in Medical Sciences Research Center, Shahid Beheshti University of Medical Sciences, Tehran, Iran, sbmu.ac.ir; ^2^ Proteomics Research Center, Faculty of Paramedical Sciences, Shahid Beheshti University of Medical Sciences, Tehran, Iran, sbmu.ac.ir; ^3^ Iranian Scientific Society of Minimally Invasive Gynecology, Tehran, Iran; ^4^ Department of Reproductive Biology, The Academic Center for Education, Culture and Research, Qom Branch, Qom, Iran; ^5^ Department of Biology and Anatomical Sciences, School of Medicine at Shahid Beheshti University of Medical Sciences, Tehran, Iran; ^6^ Student Research Committee, Department of Biology and Anatomical Sciences, School of Medicine at Shahid Beheshti University of Medical Sciences, Tehran, Iran

**Keywords:** adipose-derived mesenchymal stem cells, adipose-derived stem cells, microRNA, miRNA, proliferation, self-renewal, stem cells

## Abstract

Adipose‐derived stem cells (ADSCs) have garnered significant interest in regenerative medicine (RM) due to their abundant availability, ease of isolation, and ability to differentiate into various cell types. The growth of ADSCs is crucial for their therapeutic effectiveness, as it directly influences their ability to repair damaged tissues. MicroRNAs (miRNAs) are recognized as powerful regulators of gene expression and cellular functions, including proliferation, and are believed to play a role in modulating the proliferative potential (PP) of ADSCs. This review is aimed at exploring existing research on how miRNAs influence the PP of ADSCs, focusing on aspects such as the cell cycle, ADSC factors, and various signaling pathways, including STAT3, PI3K‐AKT, Hippo, Notch, Wnt/*β*‐catenin, and MAPK pathways that affect ADSC proliferation. We also examined the impact of miRNAs on the migration of ADSCs. Through a review of relevant studies, we identified several miRNAs that influence the PP of ADSCs through various mechanisms. These miRNAs interact with key signaling pathways, cell cycle regulators, and other elements related to cell proliferation, leading to both positive and negative effects on ADSC growth. Additionally, numerous studies have suggested that modifying miRNA expression levels could enhance the PP of ADSCs for therapeutic applications. This review provides valuable insights into the intricate regulatory networks involving miRNAs that govern the PP of ADSCs. A deeper understanding of the roles of miRNAs in ADSC proliferation holds promise for improving the efficacy of ADSC therapies and advancing the field of RM. Further research is warranted to elucidate the specific mechanisms by which miRNAs regulate ADSC proliferation and to identify new therapeutic targets for enhancing the regenerative potential of ADSCs. Exploring new miRNA targets, developing miRNA‐based therapies, and creating advanced delivery systems are promising avenues for future research. The role of miRNAs in regulating the PP of ADSCs presents exciting opportunities for the advancement of RM and tissue engineering.

**Summary**

Understanding the molecular mechanisms of the proliferation of adipose‐derived stem cells (ADSCs) is crucial for advancing regenerative medicine (RM). This review highlights the emerging role of microRNAs (miRNAs) as key modulators of ADSCs proliferation, offering insights into their potential as targets to enhance cell proliferation, expansion, and therapeutic efficacy. A deeper understanding of the roles of miRNAs in ADSC proliferation holds promise for improving the efficacy of ADSC therapies and advancing the field of RM. By synthesizing current research, this article underscores the importance of miRNA‐based regulation in proliferation of stem cells (SCs), ultimately contributing to more effective regenerative strategies. This work bridges a knowledge gap, emphasizing the significance of miRNAs in shaping the future of SC therapy.

## 1. Introduction

The SCs are undifferentiated cells known for their remarkable ability to self‐renew and differentiate into specialized cells in the body. They play a vital role in repair and regeneration, significantly aiding tissue growth and development throughout an individual′s life [1–3]. SCs can be categorized into three types: embryonic SCs, induced pluripotent stem cells (iPSCs), and adult SCs found in various tissues and organs. Embryonic SCs, which are obtained from early embryos, possess unlimited differentiation potential but pose ethical, legal, and political challenges, as well as issues related to immune rejection, which restrict their use in current research and clinical settings. In contrast, iPSCs offer similar benefits without the ethical dilemmas, although their genetic stability requires further investigation [4–6]. Mesenchymal stem cells (MSCs) are mature SCs obtained from various sources, including bone marrow, adipose tissue, placental tissue, amniotic fluid, and cord blood mesenchyme [7–11]. These cells exhibit strong anti‐inflammatory and immunomodulatory properties, making them suitable for transplantation into recipients without the risk of rejection. Given their availability and established cultivation techniques, MSCs are promising candidates for cell therapies and RM [12, 13]. ADSCs have attracted considerable attention in comparison with other types of SCs for their abundance, accessibility, easy isolation, and distinctive features. Sourced from adipose tissue, ADSCs are multipotent cells with the power to differentiate into various cells like adipocytes, osteoblasts, chondrocytes, myocytes, and endothelial cells [14–16]. Their self‐renewal and differentiation capabilities make them highly valuable for tissue repair. However, to achieve effective therapeutic results, a sufficient supply of these cells is necessary, which requires efficient replication [16, 17]. Proliferation is essential for the effective use of ADSCs in RM. Optimal proliferation allows for the growth of ADSC populations, creating a sufficient supply of cells for transplantation or tissue engineering. ADSCs that proliferate at higher rates can be easily expanded in the lab, providing the necessary quantities for therapeutic use. Additionally, increased proliferation boosts the effectiveness of ADSC‐based treatments. A larger number of proliferating ADSCs leads to enhanced cell functionality, better integration into target tissues, and improved tissue regeneration and recovery. To fully utilize the regenerative capabilities of ADSCs, it is important to understand the mechanisms behind their proliferation, which are influenced by various factors, including miRNAs [18–20]. MiRNAs are short, noncoding RNA molecules made up of approximately 23 nucleotides [21]. They are essential for regulating cell differentiation and growth in both animals and plants [22, 23]. By binding to messenger RNAs (mRNAs), miRNAs can affect gene expression through interactions with untranslated region sequences, leading to gene promotion, degradation, or repression [24, 25]. This ability to modulate the expression of multiple genes enables miRNAs to coordinate complex cellular processes [26]. In ADSCs, miRNAs play a critical role in controlling proliferation by affecting key signaling pathways and cell cycle regulators. They can either enhance or inhibit ADSC proliferation by targeting specific genes related to growth, survival, differentiation, apoptosis, and proliferation [21, 24, 27–29]. Dysregulation of miRNAs can significantly impact ADSC proliferation, as alterations in miRNA expression may lead to abnormal cell cycle regulation and reduced proliferative ability. Certain miRNAs, like miR‐128 [30], miR‐136 [31], miR‐21 [32], and miR‐193a‐3p [23], promote ADSC proliferation by targeting negative regulators of cell cycle progression and signaling pathways involved in cell proliferation. Conversely, miRNAs such as miR‐34a [33] target genes that control cell cycle regulation and proliferation‐related signaling pathways, serving as negative regulators of ADSCs proliferation. Additionally, miRNAs like miR‐146a [34], miR‐148a [35], miR‐92a [36, 37], and miR‐145‐5p [38] do not influence ADSC proliferation, indicating their neutral role in this process. These examples highlight the regulatory function of miRNAs in ADSC proliferation. By targeting specific genes and pathways, miRNAs help maintain a balance between cell proliferation and quiescence in ADSCs, ensuring regulated and coordinated growth. Understanding how miRNAs interact with ADSC proliferation can provide insights into the molecular mechanisms at play and may have implications for improving ADSC‐based therapies and tissue engineering strategies [39, 40]. This review seeks to summarize the existing understanding of how miRNAs influence the growth potential of ADSCs. It begins by emphasizing the significance of comprehending SCs and their various types, the process of proliferation, the formation of miRNAs, and the functions of miRNAs in the proliferation of ADSCs. The review also examines the SCs cycle, along with the factors and signaling pathways, including STAT3, PI3K‐AKT, Hippo, Notch, Wnt/*β*‐catenin, and MAPK, that affect the proliferation of ADSCs. Furthermore, it investigates the role of miRNAs in the migration, survival, and apoptosis of ADSCs. Each miRNA′s contribution to ADSC proliferation is discussed in detail. The goal of this review article is to provide a thorough overview of our current understanding of proliferative miRNAs in ADSCs, thereby enhancing the existing knowledge of SCs and potentially leading to the development of new therapeutic strategies involving SCs.

## 2. Methods

The study utilized databases such as PubMed, Google Scholar, and Scopus. We conducted our search using a mix of keywords: mirRNAs, MiR, ADSCs, adipose‐derived mesenchymal stem cells, proliferation, stem cells, and self‐renewal. The articles we focused on investigated the impact of miRNAs on the proliferation of ADSCs. Our inclusion criteria included relevance to the topic, subject matter, methodology, availability of full‐text articles, English language, and publication dates ranging from 2008 to 2025. Initially, we filtered articles based on their titles and abstracts, discarding those that were unrelated. We also excluded papers whose abstracts did not fit the theme or lacked sufficient data for a comprehensive assessment. The quality of the data was evaluated based on factors like randomization, conflict of interest, blinding, and ethical approval, with a 25% increase in the quality score for each criterion met. To minimize bias and improve the review′s quality, two reviewers independently assessed the quality of the selected articles according to our criteria. Data extraction was performed by one reviewer and confirmed by another. In the end, studies that met our objectives were included, categorized, and reported. We also examined the references of related articles to further enhance our search outcomes.

## 3. Results and Discussion

### 3.1. miRNA Biogenesis

**Figure 1:**
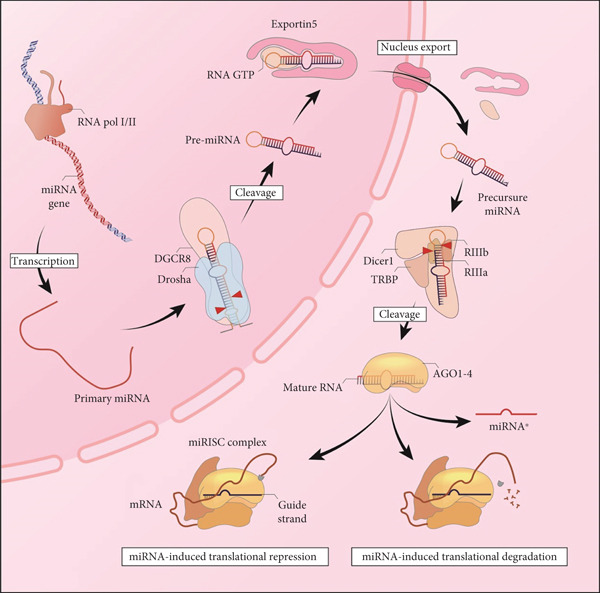
The miRNA biogenesis pathway. The miRNA production process begins with the transcription of the relevant genes by RNA polymerase II, forming an initial product called pri‐miRNA, which has a stem–loop structure. This precursor is processed in the nucleus by the Drosha‐DGCR8 complex and converted into a shorter form, pre‐miRNA. The pre‐miRNA is then transported to the cytoplasm by Exportin‐5, where the enzyme Dicer removes the loop, converting it into a double‐stranded miRNA. Finally, one of the strands enters the RISC complex as a “guide strand” and, by recognizing target mRNAs, inhibits their translation or degrades them. This pathway is under tight regulatory control at several levels.

Over the last two decades, the field of miRNA therapy has expanded significantly. The process of miRNA biogenesis is intricate and highly regulated, consisting of multiple stages and components. Any disruption in this pathway can alter gene expression and may contribute to diseases like cancer. Understanding the mechanisms of miRNA biogenesis is essential for developing new diagnostic and treatment approaches for miRNA‐related conditions. miRNAs are small noncoding RNA molecules that influence gene expression by binding to specific mRNAs, resulting in their silencing. The biogenesis of miRNAs involves a complex series of steps, including transcription, processing, and maturation. The role of miRNAs in metastasis development has prompted researchers to investigate potential therapeutic strategies [41, 42]. The standard process of miRNA production starts with RNA polymerase II transcribing miRNA genes, which are often located in intergenic areas or within the introns of protein‐coding genes (see Figure 1). The initial transcript, referred to as pri‐miRNA, is a lengthy, single‐stranded RNA molecule that can contain thousands of nucleotides. Pri‐miRNAs possess a stem–loop configuration that is recognized by the RNase III enzyme Drosha and its cofactor DGCR8, forming a microprocessor complex. This complex cleaves the pri‐miRNA at its base, resulting in a hairpin‐shaped RNA molecule called pre‐miRNA, which is generally 70–100 nucleotides long. The pre‐miRNA is then transported from the nucleus to the cytoplasm by the exportin‐5 protein. Once in the cytoplasm, the RNase III enzyme Dicer further processes the hairpin pre‐miRNAs by cutting the loop, producing a double‐stranded RNA molecule known as the miRNA duplex [43–45]. One part of this duplex, known as the guide strand, is integrated into the RNA‐induced silencing complex (RISC). This complex, which contains Argonaut proteins and other related components, is involved in identifying and silencing specific mRNAs. The guide strand attaches to the target mRNA via base pairing, resulting in either the degradation of the mRNA or the inhibition of its translation [46]. The production of miRNAs is carefully controlled through multiple levels, such as transcriptional, post‐transcriptional, and epigenetic processes. The expression of miRNA genes can be affected by transcription factors, DNA methylation, and changes in chromatin structure. Additionally, RNA‐binding proteins and other regulatory components can influence the processing and development of miRNAs [47–49].

### 3.2. Regulatory miRNAs in the Proliferation of ADSCs

Many miRNAs play a role in controlling the growth and differentiation of ADSCs. The levels of these miRNAs change throughout the differentiation process of ADSCs, and their impacts can vary based on the particular conditions and cellular surroundings [27, 50]. MiRNAs operate by targeting mRNAs to either promote their degradation or inhibit their translation, with their expression patterns differing based on the techniques used for isolating and culturing ADSCs. Table 1 provides an overview of various miRNAs linked to the growth of ADSCs. These miRNAs can influence specific genes or signaling pathways that are essential for cell cycle progression and expansion, thereby either enhancing or suppressing the proliferative ability of ADSCs. Some miRNAs have been recognized as promoters of ADSC growth, as they target genes involved in the cell cycle, DNA replication, and cell expansion, ultimately facilitating cell division and growth. Conversely, other miRNAs function as growth inhibitors by targeting genes that hinder cell cycle progression or induce cell cycle arrest. Additionally, certain miRNAs often collaborate with growth factors to modulate ADSC growth. Moreover, miRNAs can regulate pluripotency factors both directly and indirectly by targeting their genes, thus maintaining the equilibrium between self‐renewal and pluripotency in SCs. This review explores the roles of miRNAs in ADSC proliferation at various levels, including the cell cycle, stem cell factors, and signaling pathways such as STAT3, PI3K‐AKT, Hippo, Notch, Wnt/*β*‐catenin, and MAPK, which affect the proliferation potential (PP) of ADSCs. We also examine the role of miRNAs in ADSC survival, apoptosis, senescence, and migration, detailing the specific contributions of each miRNA to ADSC proliferation.

**Table 1:** This table presents miRNAs that are successful in enhancing the growth of ADSCs.

**MiRNAs**	**ADSC Source**	**Potential target gene(s)**	**Function**	**Reference**
miR‐204‐5p	Human	FOXC1	miR‐204‐5p downregulates the proliferation and osteogenic differentiation of ADSCs by decreasing the levels of FOXC1.	[51]
miR‐21	Human	TGF*β* receptor‐2	miR‐21 downregulates the proliferation and promotes adipogenic differentiation of ADSCs by decreasing the levels of TGF*β* receptor‐2 and SMAD3 level.	[52]
		SMAD3	miR‐21 inhibited the proliferation of ADSCs by acting through the SMAD3 pathway.	[21]
		PTEN and FasL	Upregulation of miR‐21 in transfected ADSCs leads to decreased apoptosis and increased proliferation through decreased PTEN and FASL expression.	[32]
miR‐1	Rat	Notch1	Overexpression of miR‐1 promotes ADSC proliferation and differentiation by regulating Notch1 and Hes1.	[22]
miR‐17	Human	BMPR2, Myc, STAT3, and E2F1‐3	miR‐17 overexpression decreases ADSC proliferation and promotes osteodifferentiation by affecting target genes.	[23]
miR‐20a	Human	FZD1/4/7, Myc, and BMPR2	miR‐20a overexpression downregulates ADSC proliferation and promotes osteogenesis by affecting target genes.	[23]
miR‐20b	Human	Not identified	Overexpression of miR‐20b decreases ADSC proliferation and promotes osteogenesis by affecting target genes.	[23]
miR‐31	Human	Not identified	Upregulation of miR‐31 inhibits ADSC proliferation and inhibits osteogenesis.	[23]
miR‐106a	Human	BMPR2, LIF, and STAT3	miR‐106a overexpression downregulates ADSC proliferation and promotes osteogenesis by affecting target genes.	[23]
miR‐125a‐5p	Human	Not identified	Upregulation of miR‐125a‐5p upregulates ADSC proliferation and decreases osteodifferentiation.	[23]
miR‐125b	Human	STAT3	Downregulation of miR‐125b upregulates ADSC proliferation and decreases osteodifferentiation by STAT3.	[23]
miR‐193a‐3p	Human	CDK6	miR‐193a‐3p regulates osteodifferentiation and cell‐cycle progression by targeting genes.	[23]
miR‐17‐92 cluster	Mouse	P21	Decreased miR‐17‐92 cluster in ADSCs increases the senescent.	[24]
miR‐26a	Rabbit	Not identified	Overexpression of miR‐26a increases ADSC viability.	[25]
	Human	CDK5	miR‐26a decreases the proliferation and adipogenic differentiation of ADSCs.	[53]
miR‐27	Human	PHB	miR‐27 overexpression restricts adipogenesis and promotes cell growth.	[26]
miR‐29b	Human	PTEN	miR‐29b upregulates osteodifferentiation and cell growth through PTEN/AKT/*β*‐catenin.	[29]
miR‐34a	Human	CDKs (2, 4, and 6), cyclins (E, D), CD44, and STAT3;	miR‐34a overexpression inhibits cell growth and promotes cellular senescence.	[54]
		RBP2, NOTCH1, and CYCLIN D1	miR‐34a promotes osteogenesis and decreases the proliferation of ADSCs.	[33]
miR‐34a‐5p	Mouse	CTRP9	Downregulation of miR‐34a raises ADSC proliferation and migration and suppresses cell apoptosis.	[55]
miR‐103a‐3p	Human	CDK6 and DICER1	Upregulation of miR‐103a‐3p suppresses ADSC proliferation and osteogenic differentiation.	[19]
	Human	SIRT7	miR‐130a‐3p overexpression promotes osteogenesis and suppresses ADSCs proliferation.	[56]
miR‐128	Porcine	p‐JNK	Overexpression of miR‐128 promotes cell proliferation and suppresses the apoptosis of porcine ADSCs.	[30]
miR‐135a‐5p	Human	MOB1B and LATS1	miR‐105a‐5p upregulation raises ADSC proliferation and adipogenic differentiation through the HIPPO pathway.	[57]
miR‐135	Rat	Not identified	miR‐135 promotes osteodifferentiation and cell growth.	[58]
miR‐136	Ovine	HSD17B12	Upregulation of miR‐136 promotes cell proliferation and inhibits adipogenesis.	[31]
miR‐137	Human	CDC42	Overexpression of miR‐137 inhibits cell proliferation and adipogenic differentiation.	[59]
		LSD1	Overexpression of miR‐137 promotes active bone formation and suppresses cell proliferation and migration.	[60]
miR‐142‐5p	Human	Not identified	miR‐142‐5p represses ADSC proliferation and reduces cell viability.	[61]
miR‐143	Rat	MAP2K5	miR‐143 overexpression regulates adipogenesis and blocks cell expansion.	[62]
miR‐143‐3p	Human	ADD3	miR‐143‐3p overexpression promotes cell proliferation and migration and improves senesces.	[63]
miR‐146a	Human	Not identified	Transfection of miR‐146a into ADSCs does not affect proliferation.	[34]
miR‐148a	Human	Not identified	miR‐148a does not affect cell cycle progression.	[35]
miR‐150	Mouse	NOTCH3	Upregulation of miR‐150 promotes adipogenesis and inhibits cell proliferation.	[64]
miR‐196a	Human	HOXC8	Upregulation of miR‐196a promotes osteodifferentiation and reduces cell proliferation.	[65]
miR‐199a‐5p	Human	SIRT1	Inhibition of miR‐199a‐5p rejuvenates aged ADSCs.	[66]
miR‐200c‐3p	Human	CD44	miR‐200c‐3p conserves stemness, contrasts senescence, and induces proliferation in ADSCs.	[67]
miR‐210	Human	PTPN2	miR‐210 promotes ADSC proliferation and migration.	[68]
miR‐221/222	Human	PTEN	miR‐221/222 promotes endothelial differentiation and ADSC proliferation and migration.	[69, 70]
miR‐302	Mouse	Oct4, Nanog, and Sox2	miR‐302 family upregulates pluripotency markers in ADSCs.	[71]
		CDKN1A and CCL5	miR‐302 promotes cell proliferation and inhibits apoptosis.	[27]
miR‐324‐5p	Rat	Not identified	miR‐324‐5p overexpression improves cell viability and migration.	[72]
miR‐335	Human	Not identified	Overexpression of miR‐335 inhibits cell proliferation and migration and osteogenic and adipogenic potential.	[73]
miR‐363	Rat	E2F3	miR‐363 overexpression inhibits ADSC mitotic clonal expansion and terminal differentiation.	[74]
miR‐375	Human	DEPTOR and YAP1	miR‐375 promotes osteodifferentiation and leads to loss of stem cell pluripotency.	[75]
miR‐378	Human	Not identified	Downregulation of miR‐378 decreases cell proliferation.	[76]
miR‐483	Human	IGF1	miR‐483‐3p promotes adipogenic differentiation of ADSCs and increases cellular senescence.	[50]
miR‐486‐5p	Human	SIRT1	miR‐486‐5p overexpression induces premature senescence and inhibits ADSCs.	[77]
miR‐503‐3p	Human	Wnt2/7	Downregulation of miR‐503‐3p promotes osteogenic differentiation and cell proliferation.	[78]
miR‐1292	Human	FZD4	Overexpression of miR‐1292 accelerated ADSC senescence and restrained osteogenesis.	[79]
miR‐1908	Human	Not identified	miR‐1908 overexpression increases cell proliferation and inhibits ADSC adipogenic differentiation.	[80]
miR‐5591‐5p	Human	AGER	miR‐5591‐5p promotes cell survival, enhances the ability of ADSCs to repair, and reduces apoptosis.	[28]
Let7c	Human	SCD1	Upregulation of let7c inhibits ADSC proliferation and osteodifferentiation.	[81]
miR‐92a	Human	Not identified	miR‐92a overexpression or downregulation does not affect ADSC viability.	[36, 37]
miR‐145‐5p	Rat	Not identified	miR‐145‐5p inhibition enhances ADSC osteogenic potential, reduces adipogenic differentiation, and promotes migration but does not affect ADSC proliferation.	[38]
miR‐429	Human	SCD1	Overexpression of miRNA‐429 inhibits ADSC proliferation and osteodifferentiation.	[82]
miR‐424	FOXO1	Human	Overexpression of miR‐424 inhibited proliferation and osteogenic differentiation.	[83]

### 3.3. miRNAs and the Cell Cycle

The cell cycle is a highly controlled process that manages cell growth, DNA replication, and cell division. It is made up of several distinct phases: G1 (first gap), S (synthesis), G2 (second gap), and M (mitosis), each with specific roles and checkpoints to ensure accurate progression through the cycle. Key miRNAs that play a role in the cell cycle primarily influence it by targeting genes involved in the transition from G1 to S phase. For instance, members of the miR‐17‐92 cluster inhibit the cyclin‐dependent kinase inhibitor (CDK) P21, thereby hindering the G1/S transition and overall cell cycle advancement [24]. MiR‐200c‐3p downregulates P21 by directly targeting it, which impacts the cell cycle [67]. An increase in miR‐34a expression leads to a decrease in various cell cycle regulators, such as CDK [22, 24, 26], that are linked to the G1/S transition. The decrease in these regulators, particularly CDK2, not only affects the cell cycle but also lowers the levels of pluripotency markers [54, 84]. Another study found that higher levels of miR‐34a were linked to the inhibition of RBP 2 and a reduction in cyclin D1 levels. RBP 2 is a crucial cell cycle regulator that binds directly to the P27 promoter [33, 85]. The absence of RBP 2 results in increased P27 levels, and combined with the reduced cyclin D1, this ultimately suppresses the proliferation of ADSCs [33]. miR‐193a‐3p and miR‐103a‐3p inhibit cell cycle progression by targeting CDK6, leading to decreased proliferation [19, 23]. MiR‐137 decreases proliferation by influencing CDC42 expression and lowering its levels [59]. CDC 42, a small GTPase, is involved in regulating the cell cycle and its transition from the G1 to the S phase by activating downstream molecules like cyclin D. miR‐302 reduces the number of cells in the G0/G1 phase and increases those in the S phase by inhibiting CDKN1A (P21) and promoting CDK2/6 and cyclin A/B genes [27].

**Figure 2:**
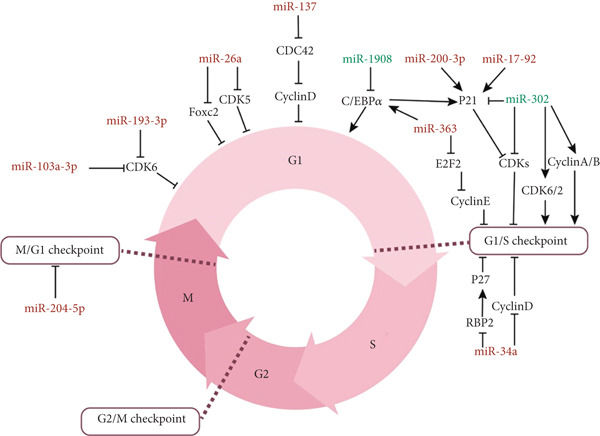
The cell cycle consists of four phases: G1, S, G2, and M, which are precisely regulated by molecular factors such as cyclins, CDKs, and inhibitory factors such as P21 and P27. By inhibiting or activating these factors, miRNAs play a role in controlling the transition between phases, especially in the transition from G1 to S phase. In this figure, the names of miRNAs inhibitory effects on cell cycle parts are shown in red, and those stimulatory effects are shown in green. These miRNAs can lead to cell cycle arrest or progression by affecting pathways such as CDK2/4/6, cyclin D1, E2F2, CDC42, and FOXC2 and regulate stem cell proliferation.

Similarly, miR‐204‐5p likely decreases ADSC proliferation by negatively regulating this process, increasing cells in the G0/G1 phase ,whereas decreasing those in the G2/M phase [51]. One of the targets of miR‐363 is the transcription factor E2F2, which plays a role in controlling the G1/S transition and directly influences Cyclin E transcription. Cyclin E is crucial for DNA synthesis and facilitates the G1/S transition. E2F2 is also a substrate for CDKs [74, 86]. In addition to promoting adipogenic differentiation, miR‐363 raises P21 levels by upregulating C/EBP‐*α* and inhibiting CDK, leading cells to enter a growth arrest phase [74]. Like miR‐363, miR‐17 reduces ADSC proliferation by affecting E2F1‐3 transcription factors [23]. Conversely, miR‐1908 has an opposing effect on key adipogenic factors, such as C/EBP‐*α*, and promotes cell cycle progression by upregulating genes that facilitate this change while decreasing the number of cells in the G1 phase [80]. miR‐26a decreases ADSC proliferation by targeting CDK5 and FOXC 2 (a key substrate of CDK5) and reducing their levels [53] (Figure 2).

### 3.4. miRNAs and Stemness Factors

Transcription factors such as OCT4, SOX2, NANOG, and KLF4 play a crucial role in preserving stem cell characteristics. miRNAs can modulate the expression of these factors. For instance, miR‐17 and miR‐20a inhibit the growth of ADSCs by affecting c‐Myc expression [23]. Similarly, miR‐150 decreases Nanog expression without impacting SOX2 and OCT4 levels [64]. On the other hand, miR‐210 enhances ADSC proliferation by elevating OCT4 and Rex 1 expression, but it does not influence Nanog, SOX2, KLF4, or c‐Myc [27]. miR‐34a significantly decreases the expression of key transcription factors such as OCT4, SOX2, NANOG, KLF4, and c‐Myc. This miRNA also contributes to the downregulation of cell cycle regulators; specifically, CDK2, which is crucial for the G1/S transition, plays a vital role in controlling the expression levels of these fundamental transcription factors [54, 84, 87]. Consequently, it can be concluded that miR‐34a diminishes the stemness and proliferation capacity of ADSCs by lowering the levels of cell cycle regulators. Similar to miR‐200c‐3p, miR‐34a also influences the stem cell marker CD44, a cell surface glycoprotein involved in Wnt signaling [54, 67, 88]. CD44 aids in the nuclear translocation of Nanog, which then forms a complex with STAT3 to activate pluripotency regulators. By decreasing CD44 levels, miR‐34a exacerbates the reduction of Nanog, thereby diminishing the stemness of the cells [54]. In contrast, miR‐200c‐3p enhances ADSC proliferation by promoting CD44 expression and downregulating the regulators of the CD44/p‐STAT3 pathway [67]. Furthermore, miR‐106a influences the stemness and proliferation of ADSCs through LIF, a cytokine that operates via the JAK/STAT signaling pathway, ultimately activating STAT3 and regulating the expression of genes essential for maintaining pluripotency, including Oct4, Nanog, and Sox2 [23].

### 3.5. miRNAs and STAT3 Signaling Pathway

The STAT3 protein is involved in various cellular functions by regulating gene expression (as noted in reference [61]). When phosphorylated, STAT3 remains in the nucleus and binds to DNA, influencing the transcription of numerous genes that control growth and cell division (proliferation) across different cell types and conditions [63]. Several miRNAs have been found to modulate the proliferation of ADSCs by impacting STAT3′s expression and activity. For instance, miR‐21 directly interacts with the 3 ^′^ end of the Stat3 gene, leading to a decrease in its expression [21]. Likewise, miR‐34a has been shown to lower both the expression and phosphorylation of STAT3 [54]. Additionally, miR‐17 and miR‐106a may also reduce STAT3 expression, thereby inhibiting ADSC proliferation through similar pathways [23]. Notably, the transcription factor NANOG, which is crucial for maintaining pluripotency and “stemness” in SCs, can form a complex with STAT3 and activate it via the CD44/NANOG/p‐STAT3 pathway. miR‐200c‐3p has been identified to enhance the expression of CD44 and p‐STAT3, thereby promoting ADSC proliferation by targeting the negative regulators of this pathway [67]. Similarly, miR‐125a‐5p has been shown to positively influence ADSC proliferation by upregulating STAT3 [23].

### 3.6. miRNAs and the PI3K‐AKT Signaling Pathway

The PI3K‐AKT signaling pathway is an intracellular pathway crucial for various cellular functions, particularly cell proliferation. It starts with the activation of phosphoinositide 3‐kinase (PI3K), which phosphorylates phosphatidylinositol‐4, 5‐bisphosphate (PIP2) to form phosphatidyl 3, 4, 5‐triphosphate (PIP3). PIP3 serves as a binding site for AKT, which is then phosphorylated by its kinase activity [32]. The phosphorylation of AKT activates multiple downstream targets that promote cell proliferation. Additionally, the PI3K‐AKT pathway activates mTOR, a vital regulator of cell growth and proliferation. PTEN acts as a negative regulator of both AKT and PIP3 and is involved in the phosphorylation of PIP3 [89]. By reducing PTEN levels, miR‐21 influences the PI3K‐AKT signaling pathway, resulting in increased proliferation of ADSCs [32]. Similarly, miR‐221/222 suppresses PTEN expression and activates the PI3K‐AKT‐mTOR pathway by enhancing the expression of phosphorylated PI3K, AKT, and mTOR [69, 70]. miR‐29b also modulates and increases the proliferation of ADSCs through the PTEN/AKT/*β*‐catenin signaling pathway [29]. mTORC1, a downstream effector of the PI3K‐AKT pathway, regulates protein synthesis, cell growth, and proliferation by phosphorylating targets like ribosomal protein S6 kinase (S6K). By activating S6K through mTORC1, miR‐375 induces negative feedback inhibition of IRS‐1‐PI3K‐AKT, leading to a decrease in AKT phosphorylation and affecting ADSC proliferation [75].

### 3.7. MiRNAs and the Hippo Signaling Pathway

Research has indicated that the Hippo signaling pathway plays a significant role in the expansion and proliferation of ADSCs. Key components of the Hippo pathway include MST1/2, MOB1, and the downstream transcriptional activators YAP and TAZ [90]. When the Hippo pathway is active, it phosphorylates and inhibits YAP and TAZ, preventing their movement into the nucleus and the activation of genes that promote cell proliferation and survival. This inhibition results in suppressed growth and decreased cell proliferation [90–92]. Additionally, miRNAs are involved in regulating the Hippo pathway, which can influence ADSCs proliferation. For instance, miR‐135a‐5p targets and inhibits negative regulators of the Hippo pathway, such as LATS1 and MOB1B, leading to enhanced YAP/TAZ activity and increased proliferation of ADSCs [57]. Conversely, miR‐375 modulates the Hippo pathway by decreasing YAP levels, which in turn reduces ADSCs proliferation [75].

### 3.8. miRNAs and the Notch Signaling Pathway

The Notch pathway influences the growth of ADSCs by activating downstream target genes that are crucial for cell cycle progression. Various miRNAs have been discovered that can regulate ADSC proliferation by interacting with components of the Notch pathway. For instance, miR‐1 has been found to inhibit ADSC growth by directly targeting Notch1 and its downstream effector, Hes1. By lowering the levels of Notch1 and Hes1, miR‐1 reduces the transcription of genes associated with cell cycle progression, thereby suppressing ADSC proliferation [22]. Additionally, other miRNAs can enhance ADSC proliferation by targeting the negative regulators of the Notch pathway. For example, miR‐21 boosts ADSC proliferation by targeting PTEN, a negative regulator of the PI3K‐AKT pathway that can indirectly inhibit Notch signaling. By downregulating PTEN, miR‐21 activates the PI3K‐AKT pathway, which subsequently promotes Notch signaling and ADSC proliferation [32]. Furthermore, some miRNAs can directly target Notch receptors or ligands, impacting ADSC proliferation. For instance, miR‐34a and miR‐150 have been shown to reduce the expression of Notch1 and Notch3 receptors, respectively, resulting in decreased Notch signaling and lower ADSC proliferation [33, 64].

### 3.9. mirRNAs and the Wnt/*β*‐Catenin Signaling Pathway

The Wnt‐*β*catenin signaling pathway is crucial for various biological functions, including cell growth, and it positively influences the self‐renewal of ADSCs [73]. In the absence of Wnt signaling, *β*‐catenin undergoes phosphorylation by a degradation complex, leading to its degradation by the proteasome. However, when Wnt ligands attach to their receptors, a signaling cascade is activated that inhibits the degradation complex, allowing *β*‐catenin to accumulate. This *β*‐catenin then moves into the nucleus, where it interacts with TCF/LEF transcription factors, activating genes that promote cell proliferation and survival [93, 94]. Several miRNAs have been found to regulate the Wnt‐*β*catenin pathway and can influence the proliferation of ADSCs. For instance, miR‐20a decreases ADSC proliferation by targeting Fez receptors (FZDs) 1/4/7, which are essential for Wnt signal transduction, leading to their downregulation [23]. miR‐503‐3p acts as a negative regulator of the Wnt pathway by targeting Wnt2/7b [78]. SIRT7, a member of the sirtuin family, interacts with *β*‐catenin, facilitating its nuclear translocation, and also interacts with TCF/LEF transcription factors, thereby affecting the expression of genes related to ADSC proliferation. Additionally, miR‐130a‐3p regulates the osteogenic differentiation and proliferation of ADSCs by targeting and reducing SIRT7 [56]. Let7c inhibits ADSC proliferation by downregulating SCD1, which is involved in the nuclear translocation of *β*‐catenin; its inhibition effectively shuts down Wnt signaling [81, 95].

### 3.10. miRNAs and MAPK Signaling Pathway

The MAPK (mitogen‐activated protein kinase) signaling pathway is a widely recognized pathway that plays a crucial role in regulating cell proliferation. It involves a series of kinase cascades, including ERK, and is activated through the phosphorylation of upstream kinases like MAP3Ks (MAPK kinase kinase) and MAP2Ks (MAPK kinase). miRNAs can influence the proliferation of ADSCs by targeting components of the MAPK signaling pathway. For instance, downregulation of miR‐34a‐5p has been reported to enhance ADSC proliferation and migration by activating the ERK1/2 pathway [55]. This suggests that endogenous miR‐34a‐5p acts as a suppressor of this pathway. [55]. Conversely, miR‐137 reduces the expression of CDC 42, which is essential for cell cycle progression from the G1 to S phase via ERK1/2 activation [59, 96]. The elevated levels of miR‐143 inhibit the expression of MAP2K5 [62], which is the upstream kinase for ERK5 and is responsible for its direct phosphorylation. ERK5 is crucial for cell proliferation and the progression of the cell cycle; its reduction halts clonal expansion [97, 98]. High levels of miR‐200c‐3p likely enhance the confluency and colony‐forming capacity of ADSCs by activating P‐ERK and concurrently downregulating P21. In epithelial cancer cells, increased miR‐200c‐3p expression promotes kRAS expression by binding to Domain 3 of the PASSF2 gene, which negatively regulates kRAS. This interaction activates the MAPK/ERK pathway, particularly P‐ERK, via kRAS [99]. In ADSC cells, miR‐200c‐3p similarly influences P‐ERK through this mechanism, boosting their proliferation [67]. Additionally, the MAPK signaling pathway can activate certain miRNAs. Once phosphorylated in the nucleus, activated MAPKs regulate various transcription factors, including Elk‐1 [100, 101]. Elk‐1 enhances its expression by binding to specific sequences in the promoter regions of the miR‐210 regulatory gene. Oxidative stress also elevates miR‐210 expression by activating the ERK pathway [68]. By downregulating PTPN 2, a negative regulator of the MAPK pathway, miR‐210 contributes to the strengthening and promotion of this pathway, thereby increasing the proliferation of ADSCs [68, 102].

### 3.11. miRNAs and the TGF‐*β* Signaling Pathway

miRNAs play a crucial role in regulating the proliferation of ADSCs by targeting essential genes in the TGF‐*β* signaling pathway. Members of the TGF‐*β* family attach to a combination of Type I and II serine‐threonine kinase receptors [27, 103]. When a ligand binds to its receptor, it triggers the TGF‐*β* signaling pathway and initiates a series of signaling events. Specifically, miR‐21 targets TGF‐*β*R 2, leading to a decrease in both its protein and mRNA levels, which in turn diminishes the TGF‐*β* signaling pathway and reduces the proliferation of ADSCs [52].

### 3.12. The Role of miRNAs in ADSC Survival and Apoptosis

miRNAs are crucial in regulating the survival and apoptosis of ADSCs. Various miRNAs have been recognized for promoting the survival of ADSCs by targeting pro‐apoptotic factors or modulating anti‐apoptotic factors. For instance, miR‐21 has been shown to prevent apoptosis in ADSCs and enhance their survival by targeting PTEN and FASL. The interaction of FASL with its receptor triggers apoptosis [32]. FKHRL promotes the expression of pro‐apoptotic genes like FASL and inhibits the PI3K‐AKT pathway (64 of 9). Once AKT function is neutralized through PTEN activation, FKHRL, similar to FASL, moves to the nucleus and initiates apoptotic signaling. When FASL interacts with its receptor, it forms the DISC, which activates caspase 8, leading to the activation of the effector caspase 3. If caspase 8 is insufficient to trigger cell death, the mitochondrial pathway is activated, regulating caspase 9, which then activates caspase 3 [32, 104]. miR‐34a inhibits apoptosis by upregulating CTRP9, which plays a role in preventing cell death [55, 105]. Similarly, miR‐302 protects against oxidative stress‐induced cell death by influencing CCL5 [27]. The JNK pathway is crucial in regulating apoptosis, with miR‐128 inhibiting apoptosis by lowering p‐JNK levels and suppressing JNK activity. The translocation of JNK protein from the cytoplasm to the nucleus activates c‐Jun, which regulates the expression of apoptosis‐related genes like Bax and Bcl2 [30, 106]. Additionally, miR‐5591‐5p reduces ROS production and apoptosis by affecting the JNK pathway and inhibiting c‐Jun signaling, thereby enhancing cell survival [28]. miR‐26a and miR‐324‐5p positively influence the survival of ADSCs, whereas miR‐142‐5p negatively impacts their survival [25, 72]. However, further research is needed to fully understand the mechanisms of these miRNAs and their effects.

### 3.13. miRNAs Influence the Aging Process in ADSCs

Cellular senescence refers to a stable halt in the cell cycle that can be triggered by various stressors, including telomere shortening and oxidative stress [107]. Research indicates that miRNAs play a crucial role in regulating pathways associated with senescence and in the development of the senescent phenotype in ADSCs. Certain miRNAs have been identified as targeting important regulators of cellular senescence, such as p16, p53, p21, and SIRT1, thereby affecting the initiation and progression of senescence [67, 77]. SIRT1, a DNA‐dependent deacetylase, shows a decrease in expression correlated with the number of passages of ADSCs [108]. For instance, miR‐486‐5p reduces SIRT1 expression by binding to its 3 ^′^ untranslated region, leading to a premature senescence‐like phenotype and reduced cell proliferation [77]. Additionally, miR‐200c‐3p and miR‐143‐3p have been reported to target the p53/p21 pathway, promoting cellular senescence and rejuvenation [63, 67], whereas miR‐483 induces premature senescence by affecting the IGF1 and Hippo pathways [50]. Conversely, research has indicated that the overexpression of miR‐1292 speeds up the senescence of ADSCs and inhibits their osteogenic potential, primarily through the Wnt/*β*‐catenin signaling pathway [79]. Moreover, additional studies have shown that miR‐17‐92 can influence ADSC senescence by altering the levels of the well‐known senescence protein p21 and affecting oxidative balance [24]. Specifically, miR‐17 inhibits the expression of thioredoxin‐interacting protein (TXNIP), which negatively regulates thioredoxin (TRX) [24, 109]. When miR‐17 is downregulated in senescent adipose‐derived mesenchymal stem cells (AMSCs), TXNIP levels rise, leading to an imbalance in redox status and reduced TRX activity. The overexpression of miR‐17 and miR‐20a, two important mirRNAs from the miR‐17‐92 cluster, can counteract the senescent characteristics of ADSCs and enhance their therapeutic capabilities in cases of acute liver failure [24]. Lastly, the level of miR‐34a increases with the number of cell passages, indicating it may contribute to the cellular senescence phenotype by regulating the production of potent senescence‐associated cytokines IL‐6 and IL‐8, although the exact regulatory mechanisms remain unclear [54, 110].

### 3.14. The Function of miRNAs in the Migration of ADSCs

miRNAs have a significant impact on cell migration. Studies indicate that the overexpression of miR‐335 reduces the migration of ADSCs influenced by interferon‐gamma (IFN‐*γ*) signaling [73]. IFN‐*γ* plays a crucial role in regulating various activities of MSCs, including their migration. It acts as a signal for tissue damage, leading to the suppression of miR‐335 in MSCs, which subsequently allows the target genes of miR‐335 that are involved in MSC migration to be expressed [73, 111]. The regulation of miRNA expression and activity in response to both internal and external signals allows cells to adjust their migratory behavior according to changing environmental conditions. The downregulation of miR‐34a‐5p enhances the migration of ADSCs by increasing the expression of CTRP9 (C1q/tumor necrosis factor‐related protein‐9) through the activation of the ERK1/2‐MMP‐9 pathway [55]. miR‐143‐3p is involved in regulating the migration of ADSCs by directly targeting and reducing the levels of ADD3, a cytoskeletal protein that inhibits cell movement. When miR‐143‐3p is overexpressed in ADSCs, it leads to an increase in the migration‐related protein fibronectin 1 (FN1), which enhances the migratory ability of these cells [63, 112]. The upregulation of miR‐210 expression, similar to miR‐143‐3p, plays a role in the migration of ADSCs. When PTPN2, a target of miR‐210, is overexpressed, it reduces the migration of ADSCs induced by hypoxia. This indicates that miR‐210 may downregulate PTPN2, leading to enhanced migration of ADSCs. Additionally, the generation of reactive oxygen species (ROS) from various sources, including mitochondrial ROS donors and PDGF‐BB, can trigger the expression of miR‐210, which subsequently promotes the migration of ADSCs [68]. To provide a comprehensive overview, the specific mirRNAs, their target genes, and their regulatory effects on ADSC proliferation through various signaling pathways are summarized in Table 2.

**Table 2:** Summary of miRNAs regulating the proliferation of adipose‐derived stem cells (ADSCs) via key signaling pathways.

**Signaling pathway**	**miRNA**	**Target/mechanism**	**Effect on proliferation**	**Species**	**Ref.**
STAT3	miR‐21	Direct binding to STAT3 3 ^′^‐UTR	↓ Inhibits	Human	[21]
	miR‐34a	↓ STAT3 expression and phosphorylation	↓ Inhibits	Human	[54]
	miR‐17, miR‐106a	↓ STAT3 expression	↓ Inhibits	Human	[23]
	miR‐200c‐3p	Targets neg. regulators of CD44/p‐STAT3	↑ Promotes	Human	[67]
	miR‐125a‐5p	↑ STAT3 expression	↑ Promotes	Human	[23]

PI3K/AKT	miR‐21	Targets PTEN	↑ Promotes	Human	[32]
	miR‐221/222	↓ PTEN → ↑ p‐AKT/p‐mTOR	↑ Promotes	Human	[69, 70]
	miR‐29b	Modulates PTEN/AKT/*β*‐catenin axis	↑ Promotes	Human	[29]
	miR‐375	Negative feedback on IRS‐1‐PI3K‐AKT	↓ Inhibits	Human	[75]

Hippo	miR‐135a‐5p	↓ LATS1, MOB1B → ↑ YAP/TAZ	↑ Promotes	Human	[57]
	miR‐375	↓ YAP1 levels	↓ Inhibits	Human	[75]

Wnt/*β*‐catenin	miR‐20a	Targets FZD 1/4/7	↓ Inhibits	Human	[23]
	miR‐503‐3p	Targets Wnt2, Wnt7b	↓ Inhibits	Human	[78]
	miR‐130a‐3p	↓ SIRT7 → impaired *β*‐catenin translocation	↓ Inhibits	Human	[56]
	Let‐7c	↓ SCD1 → impaired *β*‐catenin nuclear translocation	↓ Inhibits	Human	[81]
	miR‐1292	Targets FZD4 (induces senescence)	↓ Inhibits	Human	[79]

Notch	miR‐1	Targets Notch1, Hes1	↓ Inhibits	Rat	[22]
	miR‐21	↓ PTEN (indirectly activates Notch)	↑ Promotes	Human	[32]
	miR‐34a	↓ Notch1 receptor	↓ Inhibits	Human	[33]
	miR‐150	↓ Notch3 receptor	↓ Inhibits	Mouse	[64]

MAPK/ERK	miR‐34a‐5p	Activates ERK1/2 pathway	↓ Inhibits	Mouse	[55]
	miR‐200c‐3p	↑ p‐ERK via RASSF2/kRAS axis	↑ Promotes	Human	[67]
	miR‐210	↓ PTPN2 (negative regulator of ERK)	↑ Promotes	Human	[68]
	miR‐137	↓ CDC42 (upstream of ERK1/2)	↓ Inhibits	Human	[59]
	miR‐143	↓ MAP2K5 → ↓ ERK5	↓ Inhibits	Rat	[62]

TGF‐*β*	miR‐21	Targets TGF‐*β*R2	↓ Inhibits	Human	[52]

JNK	miR‐128	↓ p‐JNK (inhibits apoptosis)	↑ Promotes	Porcine	[30]
	miR‐5591‐5p	↓ JNK/c‐Jun (inhibits apoptosis)	↑ Promotes	Human	[28]

*Note:* Arrows indicate the direction of regulation (↑: Increase/Activation, ↓: Decrease/Inhibition). miR‐21 exhibits context‐dependent effects, promoting proliferation via the PI3K/AKT pathway while inhibiting it through STAT3 and TGF‐*β* signaling.

## 4. Conclusion

The intricate connection between miRNAs and the PP of ADSCs is a fascinating area of research with significant implications for tissue engineering. This systematic review has explored the regulatory mechanisms by which miRNAs affect ADSC proliferation, influencing cell cycle progression, stem cell factors, and various signaling pathways, including STAT3, PI3K‐AKT, Hippo, Notch, Wnt/*β*‐catenin, and MAPK pathways, all of which play a role in ADSC proliferation and migration. The studies analyzed highlight the vital function of specific miRNAs in modulating the proliferative capacity of ADSCs by targeting key signaling pathways, cell cycle regulators, and other factors related to proliferation. Abnormal expression of miRNAs has been associated with numerous diseases and conditions, underscoring the importance of understanding how miRNAs regulate ADSC proliferation. Future research holds promise for deepening our understanding of the role of miRNAs in ADSC proliferation and could pave the way for innovative therapeutic strategies. Investigating new miRNA targets, developing miRNA‐based therapies, and creating advanced delivery systems are exciting avenues for further exploration.

## 5. Future Opportunities

The role of miRNAs in regulating the PP of ADSCs presents exciting opportunities for advancements in RM and tissue engineering. As research progresses, several potential avenues may emerge:

1. Identification of new miRNA targets: Further exploration of the miRNA landscape in ADSCs could uncover new miRNAs that play a vital role in regulating ADSC proliferation. Comprehensive profiling and functional analyses may help identify additional miRNA targets and signaling pathways that affect ADSC growth.
2. Therapeutic targeting of miRNAs: The development of miRNA‐based therapies holds promise for specifically enhancing the PP of ADSCs. Future studies could focus on creating miRNA mimics or inhibitors to modify miRNA expression levels and boost ADSC proliferation for therapeutic applications.
3. Creation of miRNA delivery systems: Efficiently delivering miRNAs to ADSCs is crucial for harnessing their PP. Future research may prioritize the development of innovative delivery methods, such as nanoparticles or viral vectors, to facilitate effective and targeted miRNA delivery to ADSCs in living organisms.
4. Combination therapies: Merging miRNA‐based strategies with other regenerative approaches, such as growth factors or scaffold technologies, could improve the regenerative capabilities of ADSCs. Future investigations could explore the synergistic effects of miRNA modulation alongside other regenerative methods to enhance therapeutic outcomes.
5. Clinical application: Translating findings from preclinical studies to clinical practice represents a significant future opportunity in the regulation of ADSC proliferation by miRNAs. Clinical trials evaluating the safety and efficacy of miRNA‐based therapies for enhancing ADSC proliferation could pave the way for new regenerative treatments.

## Conflicts of Interest

The authors declare no conflicts of interest.

## Author Contributions

The author contributions for the project are as follows: A.M. and M.B.: They wrote and drafted the paper. A.M. and H.A.: They added critical comments. K.B.: She edited the manuscript for content and grammar. A.M.: He planned the project and communicated with the journal.

## Funding

No funding was received for this manuscript.

## Data Availability

Data sharing is not applicable to this article as no datasets were generated or analyzed during the current study.
